# *Tongoloa arguta* (Apiaceae), a new species from southwest China

**DOI:** 10.3897/phytokeys.164.54927

**Published:** 2020-10-21

**Authors:** Ling-Jian Gui, Jun Wen, Yan-Ping Xiao, Ting Ren, Hong-Yi Zheng, Xing-Jin He

**Affiliations:** 1 Key laboratory of Bio-Resources and Eco-Environment of Ministry of Education, College of Life Sciences, Sichuan University, 610065, Chengdu, Sichuan, China Sichuan University Chengdu China

**Keywords:** Apiaceae, China, new species, phylogeny, *
Tongoloa
*

## Abstract

A new species *Tongoloa arguta* (Apiaceae) is described and illustrated in this article. The new species grows in alpine bushes and meadows in south-western China. It resembles *T. silaifolia*, but differs from the latter by the length of the stem, ultimate segments of leaf and rays of the umbel. Phylogenetic analysis, based on nuclear ribosomal DNA internal transcribed spacer (ITS) sequences, is provided, as well as comparative morphology between related species.

## Introduction

*Tongoloa* H.Wolff (Apiaceae) is a genus comprising about 15–20 species distributed mainly in southwest China, with a few species extending westwards to central Nepal (Watson 1999; Pan and Watson 2005; Zhou et al. 2009). *Tongoloa* species are characterised by having conic taproots, inflated and membranous leaf sheaths, cordate fruit base and filiform fruit ribs (Wolff 1925; Mukherjee and Constance 1991; Pimenov and Kljuykov 2000; Pan and Watson 2005). Some species have been described in *Pimpinella* L. due to the morphological similarity (Boissieu 1902, 1906). The genus *Tongoloa* was formally established by Wolff (1925) and accepted as an independent genus (Pimenov and Kljuykov 1995; Pimenov 2017). Molecular phylogenetic analyses, based on limited materials of nuclear ribosomal DNA internal transcribed spacer (ITS) and chloroplast markers, indicated that *Tongoloa* is part of the East Asia clade of Apioideae (Apiaceae) (Zhou et al. 2009; Downie et al. 2010).

So far, 15 species of *Tongoloa* have been identified from different regions of China (Pimenov 2017), most of which being known from the Hengduan Mountains. While studying specimens in herbariums (CDBI, PE), we noticed several interesting specimens of *Tongoloa* collected from Sichuan and Yunnan, which have short stems and fewer rays of the umbel (3–8). Through field investigation and anatomical study, we confirmed that this species does not match any previously-published description of *Tongoloa* found from southwest China to central Himalaya. Further molecular analysis revealed significant differences between this species and its relatives. The results allow us to infer that these newly-collected specimens from Sichuan and Yunnan belong to a new species.

## Materials and methods

We collected an unknown *Tongoloa* species from several populations in Yunnan and Sichuan Provinces. In addition to the samples collected in the field, the type specimens of *Tongoloa* and high-resolution type specimen photos were examined, including the specimens deposited in K, P, E, B, A, GB, LD, MW, NY, GH, W, US, PE, KUN, CDBI, WUK and HNWP. Considering the similarity between the new species and *T. silaifolia*, as well as other related species, we compared their morphological characteristics. The fresh fruits were preserved with formaldehyde-acetic acid-alcohol (FAA) for anatomical study. The mericarp transverse sections were examined using a stereomicroscope (Nikon SMZ25, Japan) after safranin O-fast green staining.

A plant genomic DNA kit (CWBIO, China) was used to extract total DNA from silica-dried leaves. Referring to the previous studies (White et al. 1990; Zhou et al. 2009), we used nuclear ribosomal DNA internal transcribed spacer (ITS) sequences for phylogenetic inference. Amplification was undertaken using a volume of 30 µl with 15 µl 2 × Taq MasterMix (CWBIO, China), 10 µl ddH_2_O, 1.5 µl forward primer, 1.5 µl reverse primer and 2 µl total DNA. The PCR reaction was performed in Geneamp PCR System 9700 (USA) with initial denaturation at 95 °C for 2 min, 35 cycles of 94 °C for 60 s, 52.5 °C for 45 s and 72 °C for 60 s and a final extension of 72 °C for 7 min. PCR products were sent to BGI (China) for sequencing. The GenBank accession numbers and sample information of the ITS sequences used in this study are shown in Table 1.

To determine the systematic position of the new species, 37 ITS sequences with accession numbers were obtained from GenBank, including 9 species of *Tongoloa* (Table 1). Taxa of *Chamaesium* clade were selected as the outgroup (Downie et al. 2010). Maximum Likelihood (ML) analyses with GTR + G + I model and 1000 bootstrap (BS) replicates was performed using MEGA7 (Kumar et al. 2016). Bayesian Inference (BI) analysis was conducted with MrBayes version 3.2 (Ronquist et al. 2012) and the Markov Chain Monte Carlo (MCMC) search was performed for 1 × 10^8^ generations.

**Table 1.** Taxa and voucher information of the used ITS sequences.

Taxon	Locality	Voucher information	GenBank number
*Bupleurum chinense*	China, Anhui, Dabieshan	CB Wang 09017 (SZ)	GU570615
*Bupleurum gibraltaricum*	Spain, Sevilla	S.S. Neves 35 (E)	AF479851.1
*Bupleurum tenuissimum*	Portugal, Beira Litoral	S.S. Neves 22 (E)	AF481932.1
*Chamaesium paradoxum*	China, Sichuan, Daocheng-Litang	ZJ0560 (KUN)	EU236161.1
*Chamaesium thalictrifolium*	China, Sichuan, Zhangla-Caowan	ZJ0607 (KUN)	EU236162.1
*Chamaesium wolffianum*	China, Yunnan, Shudu Lake	ZJ0525 (KUN)	EU236163.1
*Changium smyrnioides*	China, Jiangxi, Jiujiang, Pengze	PZ2 (NAS)	HQ185254.1
*Chuanminshen violaceum*	China, Sichuan, Cangxi, Xinlong nursery	J105 (KUN)	FJ385040.1
*Cyclorhiza peucedanifolia*	China, Yunnan, YuLong, Daju, Xiahutiao	J034 (KUN)	FJ385042.1
*Cyclorhiza waltonii*	China, Sichuan, Derong	ZJ0536 (KUN)	EU236165.1
*Hansenia forbesii*	China	QH6	KJ999463.1
*Hansenia oviformis*	China, Qinghai, Maqin	H43 (WNU)	MF787544.1
*Hansenia weberbaueriana*	China, Yunnan, KIB nursery	ZJ0697 (KUN)	EU236180.1
*Haplosphaera phaea*	China, Yunnan, Shudu Lake	ZJ0521 (KUN)	EU236167.1
*Heptaptera anisoptera*	Iran, Lorestan	Pimenov et al. 438 (MW)	AY941273.1, AY941301.1
*Hymenidium nanum*	Kirghizstan, Sarydzhas basin	Kozhevnikova s.n. (LE)	GQ379333.1
*Hymenolaena candollei*	India, Jammu and Kashmir	Pimenov and Kljuykov 59 (MW)	FJ469958.1, FJ483497.1
*Hymenolaena badachschanica*	Tadjikistan, Badakhshan, Andarob	Sultanov 1121 (LE)	GQ379332.1
*Hymenolaena pimpinellifolia*	Kirghizia, Kyrgyz Alatoo	Pimenov 398 (MW)	FJ469959.1, FJ483498.1
*Komarovia anisosperma*	Uzbekistan, Zeravscshtan	178(MW)	AF077897.1
*Physospermopsis delavayi*	China, Yunnan, YuLong Snow Mt.	J033 (KUN)	FJ385056.1
*Pleurospermum amabile*	China, Yunnan, Deqin, Baimaxueshan	GLJ19100605 (SZ)	MT124614
*Pleurospermum franchetianum*	China, Sichuan	YY (WNU)	KY848849.1
*Pleurospermum uralense*	China, Liaoning	LQX031 (NAS)	JF977839.1
*Pterocyclus angelicoides*	China, Xizang	G19082501(SZ)	MN689078
*Pterocyclus rotundatus*	China	G18092501-1 (SZ)	MK078059.1
*Sinolimprichtia alpina* YN	China, Yunnan, Deqin, Baimaxueshan	GLJ19100702 (SZ)	MT124613
*S. alpina* XZ	China, Xizang	0465919 (KUN)	FJ385064.1
*S. alpina* SC	China, Sichuan, Yajiang, Jianziwanshan	LH2018081402 (SZ)	MT124609
*Tongoloa arguta* YN1	China, Yunnan, Shangri-la, Daxueshan	A11 (SZ)	MT124619
*T. arguta* YN2	China, Yunnan, Deqin, Baimaxueshan	GLJ18082102 (SZ)	MT124599
*T. arguta* SC1	China, Sichuan, Yajiang, Kazilashan	GLJ18092002 (SZ)	MT124615
*T. arguta* SC2	China, Sichuan, Yajiang, Jianziwanshan	GLJ19092802 (SZ)	MT124612
*Tongoloa dunnii*	China, Hubei, Shennongjia	GLJ18091102 (SZ)	MT124601
*Tongoloa elata*	China, Sichuan, Songpan, Huangshengguan	GLJ19080404 (SZ)	MT124607
*Tongoloa loloensis*	China, Yunnan, Eryuan, Baicaoluo	GLJ18103002_1 (SZ)	MN630615
*Tongoloa stewardii*	China, Fujian, Taining, Huangyanfeng	GLJ18090802_2 (SZ)	MN630614
*Tongoloa silaifolia*	China, Chongqing, Chengkou	JQP19081607_2 (SZ)	MT124617
*Tongoloa* sp.	China, Qinghai, Yushu, Jiangxigou	GLJ19092201 (SZ)	MT124610
*Tongoloa taeniophylla*	China, Sichuan, Kangding, Paomashan	GLJ18082902 (SZ)	MT124598
*Tongoloa tenuifolia*	China, Yunnan, YuLong Snow Mt.	J075 (KUN)	FJ385066.1
*Trachydium roylei*	Pakistan, Hazara	B. Dickore, 13244	FJ469972.1, FJ483510.1
*Trachydium simplicifolium*	China, Yunnan, Lijiang, Yulongxueshan	GLJ19111401 (SZ)	MT124618
*Trachydium souliei* YN1	China, Yunnan, Deqin, Baimaxueshan	GLJ18082103 (SZ)	MT124603
*T. souliei* YN2	China, Yunnan, NW part, Degen Co.	Pimenov et al. 472 (MW)	FJ469973.1, FJ483511.1

Note: Province’s names were indicated near the manes of the species if two or several different samples were used for the molecular analysis. YN = Yunnan, SC = Sichuan, XZ = Xizang (Tibet).

## Results and discussion

### Phylogenetic analysis

**Figure 1.**
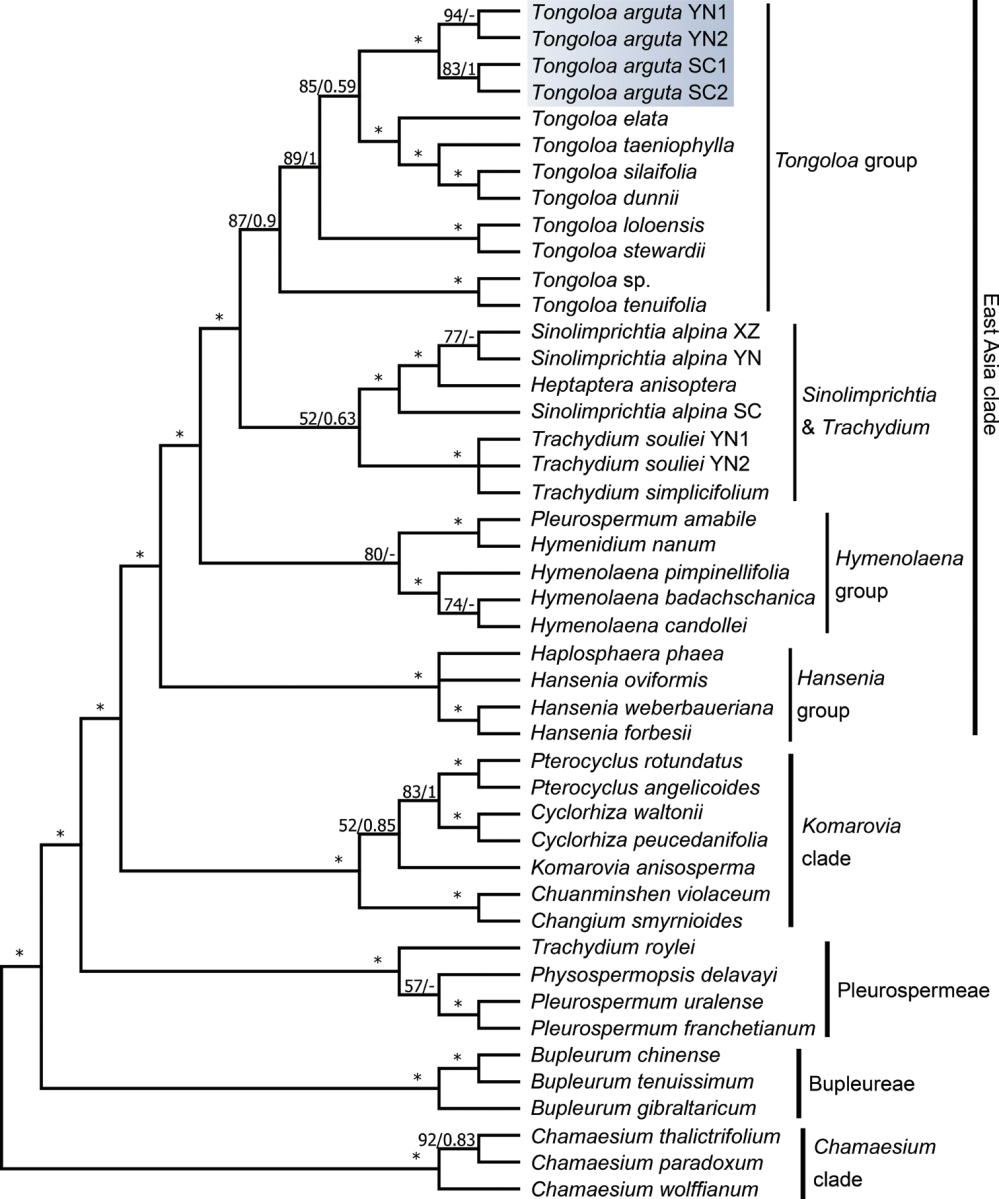
Phylogenetic tree of *Tongoloa* and related groups inferred from ITS based on ML and BI methods. MLBS / BIPP values were shown above the branches. Asterisks (*) denoted strong support (MLBS ≥ 90% and BIPP ≥ 0.90).

The total length of ITS sequence alignment with gaps was 467 bp (without 5.8S rDNA genes). *Tongoloa arguta* yielded high sequence divergence values with related species, such as *T. silaifolia* (3.2%–4.0%), *T. elata* (3.7%–4.6%) and *T. taeniophylla* (5.0%–5.5%). Analysis of the data using ML and BI methods obtained similar trees with high MLBS and BI posterior probability (PP). Phylogeny reconstruction showed that *T. arguta* positioned in the *Tongoloa* clade and different populations of this species formed a strongly-supported monophyletic group (MLBS ≥ 90% and BIPP ≥ 0.90) (Fig. 1).

These results supported *T. arguta* as an undescribed and distinct species of *Tongoloa*.

## Taxonomy treatment

### Tongoloa arguta

Taxon classificationPlantae

L.J.Gui & X.J.He sp. nov.

EBC93184-2E95-552F-A3A1-5903F41598E5

urn:lsid:ipni.org:names:77212299-1

**Figure 2.**
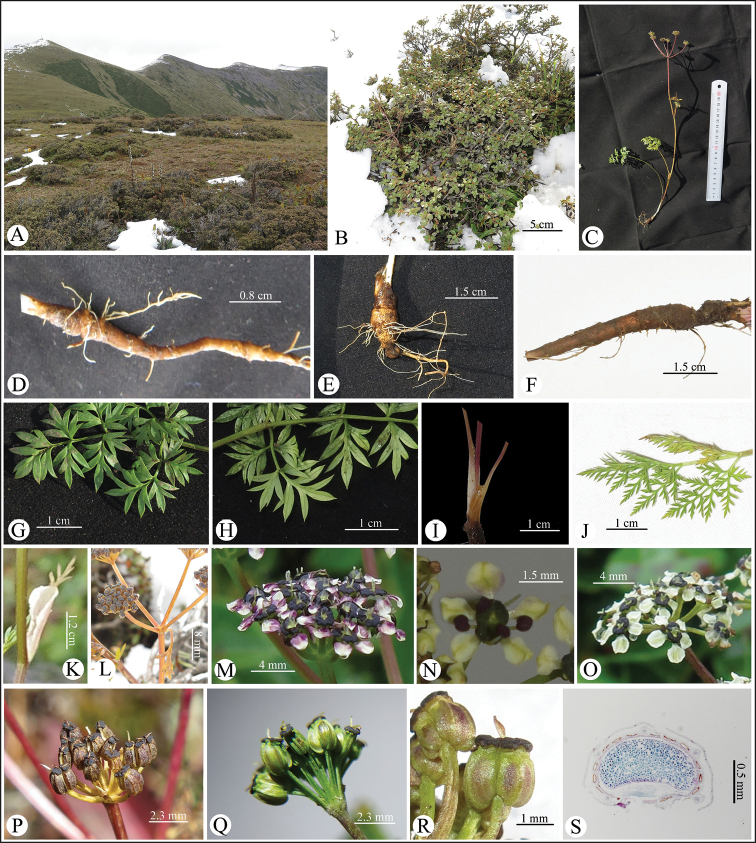
*Tongoloa arguta* sp. nov. **A** species habitat (Mt. Jianziwanshan, Sichuan, China) **B** plant in the bush surrounded by snow **C** plant **D–F** roots **G, H** basal leaf, ventral and dorsal view **I** membranous sheath of basal leaves **J** middle leaf **K** upper leaf with membranous petiole **L** umbel and bract **M–O** flowers; **P–R** fruits **S** mericarp transverse section.

**Figure 3.**
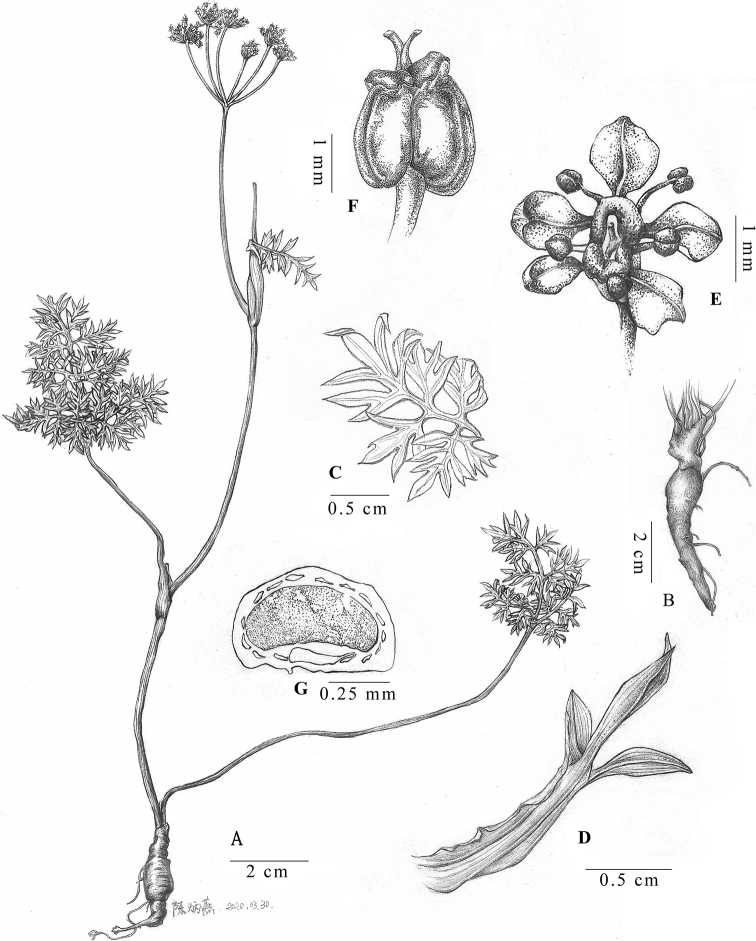
*Tongoloa arguta* sp. nov. **A** habit **B** root **C** basal leaf blade **D** leaf-like bract, only appears in some individuals **E** flower **F** fruit **G** mericarp transverse section. Drawn by Bing-yan Chen.

Figs 2 , 3 , Table 2

#### Type.

China. Sichuan: Kangding, Zheduoshan Pass, 4300 m alt., 30°4'N, 101°48'E, 26 Sep 2019, *Lingjian Gui GLJ19092601* (***holotype***: SZ).

**Table 2.** Morphological comparison between *Tongoloa arguta* and similar species.

Characters	*T. arguta*	*T. silaifolia*	*T. elata*	*T. gracilis*
Height	10–50 cm	28–60 cm	20–75 cm	25–75 cm
Root	long-conic	conic	conic	slender
Stem	purplish	purplish	purplish	purplish
Lower leaves	2–3-ternate/pinnate, ultimate segments lanceolate, 1–4 mm, apex acute	2–3-ternate/pinnate, ultimate segments linear, 5–18 mm, apex acute	3–4-ternate/pinnate, ultimate segments linear, 5–15 mm	3-ternate/pinnate, ultimate segments linear-lanceolate, 3–15 mm
Bracts	often absent, sometimes 1, leaf-like	absent	absent	absent
Bracteoles	absent	usually absent or 1–5, linear	absent	absent
Rays	3–8	8–22	6–16	5–11
Petal	apex obtuse	apex obtuse	apex obtuse-rounded	apex with incurved tips
Fruit	broadly ovoid	broadly ovoid	broadly ovoid	oblong-ellipsoid
Ribs	filiform	filiform	slender	filiform

#### Diagnosis.

*Tongoloa arguta* sp. nov. is morphologically similar to *T. silaifolia*. However, the new species can be distinguished from the latter by its short stems (10–50 cm), while *T. silaifolia* has longer ones (28–60 cm); The ultimate segments of the lower leaf of *T. arguta* are acute and short (1–4 mm), while those of *T. silaifolia* are linear and longer (5–18 mm). The umbels of *T. arguta* have 3–8 rays, which are significantly less than those of *T. silaifolia* (8–22).

#### Description.

Plants 10–50 cm. Root usually long-conic. Stem thinly ribbed, glabrous, purplish to green, branched. Leaf sheaths inflated, membranous; blade triangular in outline, 3–5 × 2–3.5 cm, 2–3-ternate/pinnate; ultimate segments lanceolate, 1–4 × 1–2 mm, apex acute. Umbels terminal or lateral; bracts often absent or sometimes 1, leaf-like, ca. 2–4 × 1 cm, bracteoles absent; rays 3–8; umbellules 13–25-flowered. Calyx teeth minute; petals obovate, white to purple, apex obtuse; stylopodium depressed, dark purple; styles short, reflexed. Fruit broadly ovoid, ca. 2 × 1.7 mm, base cordate; ribs 5, filiform; vittae 3 in each furrow, ca. 4 on commissure. Ventral surface of endosperm slightly concave to plane.

#### Etymology.

The species epithet “arguta” was given to describe the acute tips of the ultimate segments of leaves.

#### Phenology.

The species was observed flowering from August to September and fruiting from September to October.

#### Distribution and habitat.

*Tongoloa arguta* is distributed from Sichuan (Kangding, Yajing) to Yunnan (Deqin, Shangri-la) in south-western China. It grows in alpine bushes and meadow**s** from 4000 m up to 4500 m alt.

#### Additional specimens examined.

China. Sichuan: Kangding, Xinduqiao, Zheduoshan, 4000 m alt., 3 Sep 1982, *Taichang Wei 29664* (CDBI0095011); Kangding, north slope of Zheduoshan, 4000 m alt., 22 Sep 1984, *Yongjiang Li 454* (CDBI0172327); Yajiang County, Jianziwanshan, 4400 m alt., 28 Sep 2019, *Lingjian Gui & Chang Peng GLJ19092802* (SZ); Yajiang County, Kazilashan, 4400 m alt., 20 Sep 2018, *Lingjian Gui GLJ18092002* (SZ). Yunnan: Deqin County, Baimaxueshan pass, 4350 m alt., 21 Aug 2018, *Lingjian Gui GLJ18082102* (SZ); Shangri-la, Daxueshan pass, 4340 m alt., *Yanping Xiao A11* (SZ); Zhongdian, Deqen, Beima Shan, on the south side of road, 4675 m alt., 25 Sep 1994, *ACE 1287* (PE00755697).

#### Conservation status.

*Tongoloa arguta* is common in some alpine bushes and meadows at an altitude of about 4300 m in Yunnan and Sichuan, where human activities and especially yak grazing pose a potential threat to its survival. We categorise *T. arguta* as Near Threatened (NT), according to IUCN (2019).

## Supplementary Material

XML Treatment for Tongoloa arguta
